# Prognostic value of right atrial strains in arrhythmogenic right ventricular cardiomyopathy

**DOI:** 10.1186/s13244-024-01630-x

**Published:** 2024-02-27

**Authors:** Jin-Yu Zheng, Bing-Hua Chen, Rui Wu, Dong-Aolei An, Ruo-Yang Shi, Chong-Wen Wu, Lang-Lang Tang, Lei Zhao, Lian-Ming Wu

**Affiliations:** 1 Department of Radiology, Renji Hospital, School of Medicine, Shanghai Jiao Tong University, Shanghai, 200127 People’s Republic of China; 2 Department of Radiology, Longyan First Hospital, Affiliated to Fujian Medical University, Longyan, 364000 People’s Republic of China; 3 Department of Radiology, Beijing Anzhen Hospital, Capital Medical University, Beijing, 100029 People’s Republic of China

**Keywords:** Arrhythmogenic right ventricular cardiomyopathy, Arrhythmogenic right ventricular dysplasia, Magnetic resonance imaging, Right atrial function, Strain

## Abstract

**Objectives:**

Arrhythmogenic right ventricular cardiomyopathy (ARVC) is an inherited cardiomyopathy characterized by progressive fibrofatty infiltration of atrial and ventricular myocardium resulting in adverse cardiac events. Atrial function has been increasingly recognized as prognostically important for cardiovascular disease. As the right atrial (RA) strain is a sensitive parameter to describe RA function, we aimed to analyze the prognostic value of the RA strain in ARVC.

**Methods:**

RA strain parameters were derived from cardiac magnetic resonance (CMR) images of 105 participants with definite ARVC. The endpoint was defined as a combination of sudden cardiac death, survival cardiac arrest, and appropriate implantable cardioverter-defibrillator intervention. Cox regression and Kaplan–Meier survival analyses were performed to evaluate the association between RA strain parameters and endpoint. Concordance index (C index), net reclassification index (NRI), and integrated discrimination improvement (IDI) were calculated to assess the incremental value of RA strain in predicting the endpoint.

**Results:**

After a median follow-up of 5 years, 36 (34.3%) reaching the endpoint displayed significantly reduced RA strain parameters. At Kaplan–Meier analysis, impaired RA reservoir (RARS) and booster strains (RABS) were associated with an increased risk of the endpoint. After adjusting for conventional risk factors, RARS (hazard ratio [HR], 0.956; *p* = 0.005) and RABS (HR, 0.906; *p* = 0.002) resulted as independent predictors for endpoint at Cox regression analyses. In addition, RARS and RABS improved prognostic value to clinical risk factors and CMR morphological and functional predictors (all *p* < 0.05).

**Conclusion:**

RARS and RABS were independent predictors for adverse cardiac events, which could provide incremental prognostic value for conventional predictors in ARVC.

**Critical relevance statement:**

We evaluated the prognostic value of right atrial strain in ARVC patients and suggested cardiologists consider RA strain as a predictive parameter when evaluating the long-term outcome of ARVC patients in order to formulate better clinical therapy.

**Key points:**

• Patients with ARVC had significantly reduced RA strain and strain rates compared with healthy participants.

• Participants with lower RA reservoir and booster stains were associated with a significantly higher risk of adverse cardiac events.

• RA booster and reservoir strain provide incremental value to conventional parameters.

**Graphical Abstract:**

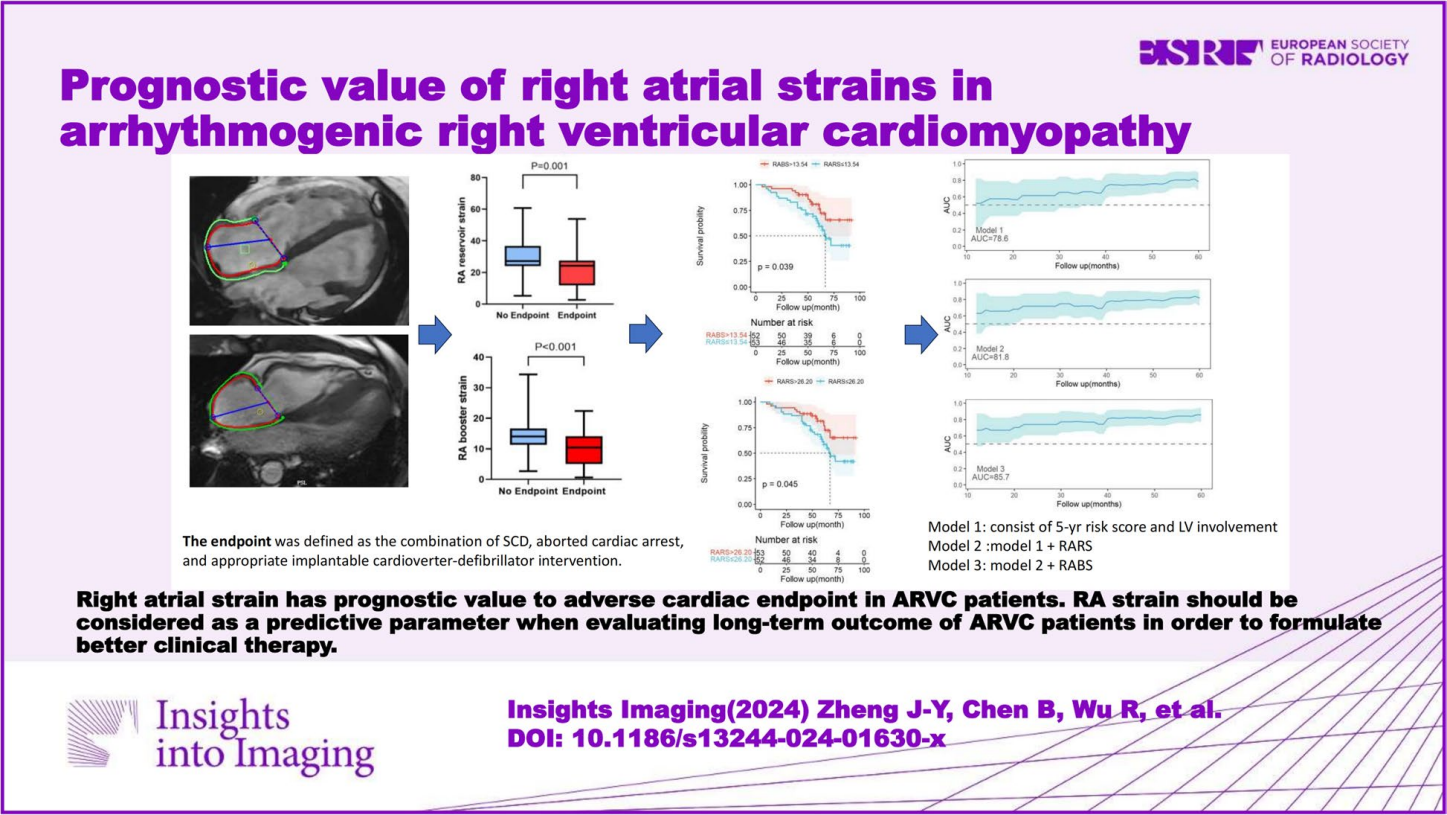

**Supplementary Information:**

The online version contains supplementary material available at 10.1186/s13244-024-01630-x.

## Introduction

Arrhythmogenic right ventricular cardiomyopathy (ARVC) is generally recognized as a genetic cardiomyopathy characterized by fatty and fibrous replacement of predominantly right ventricular (RV) myocardium [1, 2]. Left ventricular (LV) and biatrial involvement have also been detected in post-mortem examination and clinical tests [3–5]. Adverse cardiovascular events occur as ARVC progresses. Among these events, fatal ventricular arrhythmias and sudden cardiac death have been ranked as the worst clinical manifestations [6]. Hence, seeking accurate predictors for serious cardiac events is of utmost importance.

Cardiac magnetic resonance (CMR) is the preferred imaging technique for identifying ventricular structural and functional disorders with noninvasive tissue characterization in ARVC [6]. As a “one-step” examination, it offers plenty of cardiac information for clinical diagnosis. ARVC presentations on CMR, which include but are not limited to enlarged RV end-diastolic volume index (RVEDVI), decreased RV ejection fraction (RVEF), and LV involvement, have significant prognostic implications in ARVC [7, 8]. Previous studies on predictions in ARVC mainly targeted ventricular dysfunction analysis [7–11], while the discovery of atrial involvement engaged in other cardiac diseases provides another important insight into the ARVC mechanism [12, 13]. Besides ventricular functional degeneration, atrial functional disorders have been considered predictive factors for ARVC-related outcomes, especially right atrial (RA) strain [14]. RA longitudinal strain and strain rate can be evaluated in three phases differentiated by RA function, i.e., reservoir function for the systemic venous return during ventricular systole and atrial diastole, conduit function allowing blood to flow passively into the ventricle during early ventricular diastole and booster function pump blood into the right ventricle in late ventricular diastole [15]. In a prospective cohort study, RA strain in ARVC has been found to be responsible for atrial arrhythmias by CMR tissue tracking [14]. Likewise, a retrospective study based on a single-center cohort suggested RA strain impairment in ARVC patients was associated with an increased risk of cardiovascular events using speckle-tracking echocardiography [16].

While CMR-assessed RA strain is considered a strong predictor for cardiovascular events in several cardiac diseases [14, 17, 18], it remains unclear whether it is a long-term prognostic factor for predicting ventricular arrhythmias in ARVC. Accordingly, this multi-center study aimed to investigate whether RA strain parameters derived from CMR are associated with adverse cardiac events in ARVC patients and whether RA strain may add an incremental prognosis value to conventional risk factors in ARVC.

## Materials and methods

### Patients

A total of 120 participants who underwent CMR scans with definite ARVC from 3 different institutions between May 2013 and May 2020 were enrolled in this prospective study. Definite ARVC was diagnosed according to modified TFC (Task Force Criteria) only when patients met 2 major or 1 major and 2 minor or 4 minor criteria from different categories including CMR, echocardiography, and electrocardiogram [19]. In addition, exclusion criteria were defined based on contraindications for CMR examination and image quality: renal function impairment with glomerular filtration rate < 30 mL/min, arrhythmia, claustrophobia, CMR incompatible devices. We also recruited 50 age- and sex-matched healthy volunteers without any cardiac disease or other disease with cardiac involvement to give the normal standard of CMR parameters.

### CMR acquisition

CMR was performed using a 3.0 T scanner (Ingenia, Philips, Best, the Netherlands) with a 12-element phased-array coil. Short- and long-axis with two-, three-, and four-chamber cine imaging covering the whole RV with 30 frames was performed with balanced steady-state free precession (SSFP). The parameters included field of view (FOV), 300 × 300 mm^2^; voxel size, 2 × 1.75 × 6 mm^3^ (short-axis: gap, 0 mm); flip angle (FA), 45°; bandwidth, 1410 Hz/pixel; repetition time (TR), 3.20 ms; and echo time (TE), 1.60 ms. The main parameters of T2WI-STIR (T2-weighted short-tau triple inversion recovery) included FOV, 300 × 300 mm; voxel size, 1.55 × 1.9 × 10 mm^3^; flip angle, 90°; bandwidth, 980 Hz/pixel; and TR/TE, 2R-R/90 ms. The details about late gadolinium enhancement (LGE) which was acquired 10 min after administrating gadolinium with Gd-DTPA (0.15 mmol/kg, diethylenetriaminepentacetate; Bayer Schering Pharma AG, Germany) included FOV, 300 × 300 mm; voxel size, 1.8 × 1.9 × 10 mm^3^; flip angle, 25°; bandwidth, 830 Hz/pixel; and TR/TE, 4/2 ms.

### Images analysis

**Fig. 1:**
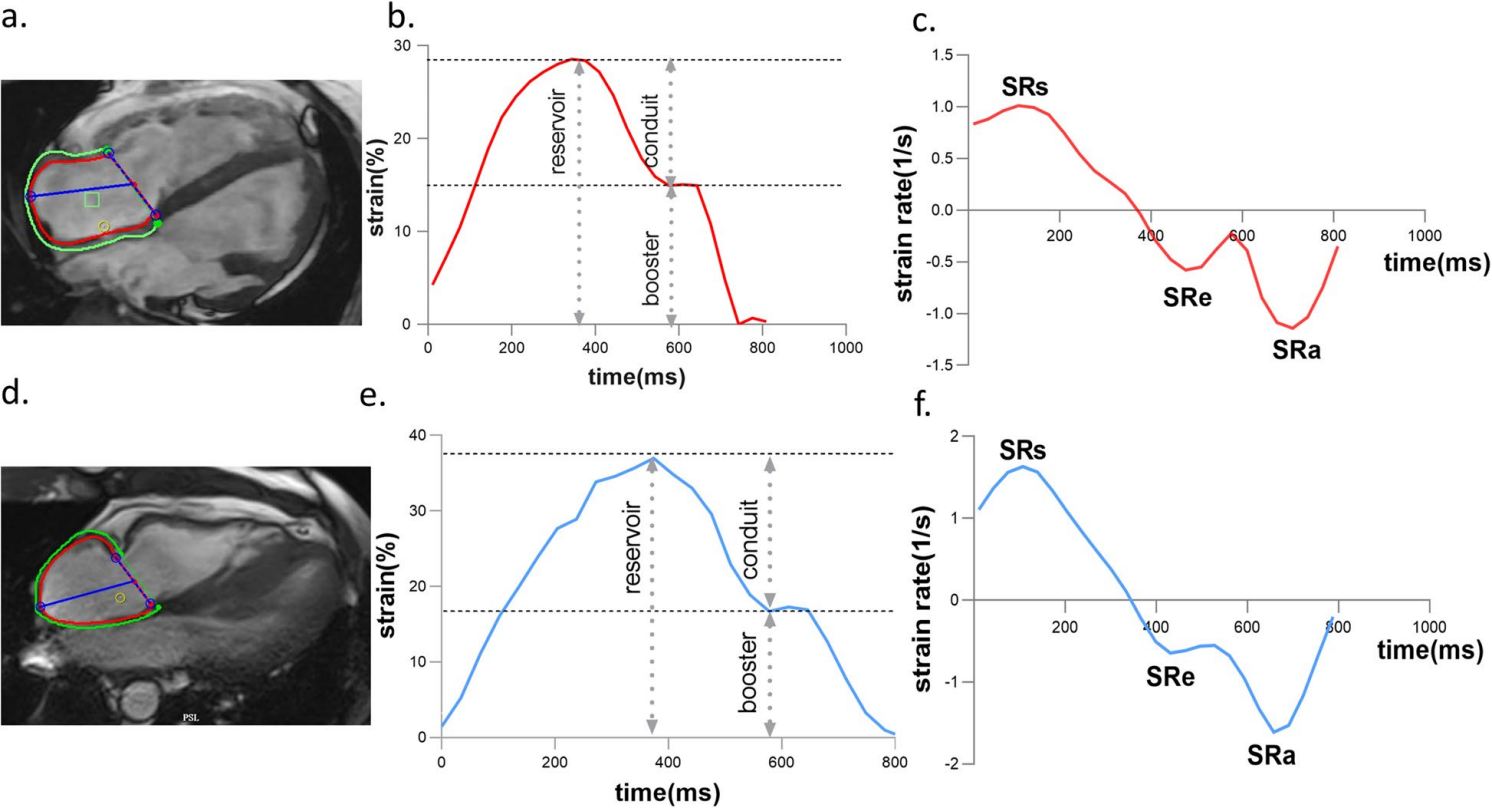
Endocardial and epicardial contour of the RA marked manually at end-diastolic phase in four-chamber long-axis views (**a**, **d**). Typical right atrial strain and strain rate curves in two definite ARVC patients with (**b**, **c**) and without (**e**, **f**) endpoint. SRs, reservoir strain rate; SRe, conduit strain rate; SRa, booster strain rate

All definite ARVC cases based on TFC diagnosis from different institutions were collected in a core laboratory. Two radiologists (W.L.M. with 12 years of experience and C.B.H. with 9 years), who were blinded to each other and clinical information, independently analyzed CMR images using dedicated commercial software Cvi42 version 5.11.3 (Circle Cardiovascular Imaging Inc., Calgary, Canada). Ventricular function and volumes were quantified from short-axis cine images, and RA function and volumes were evaluated from two- and four-long-axis cine images. End-diastolic volume and end-systolic volume were adjusted to the body surface area. RA longitudinal strain analysis was conducted only on four-chamber long-axis views. RA endocardial and epicardial contours were manually identified in the end-diastolic phase (Fig. 1). The CVI42 software automatically tracked the contours consecutively in all phases covering the whole cardiac cycle and calculated strain. The myocardial border was reviewed and manually readjusted when the contour recognized by Cvi42 was offset from the actual one so as to obtain high accuracy. RA strain, including reservoir strain, booster strain, conduit strain, and the corresponding strain rate, were the average of the results of blindly repeated tracking performed three times (Fig. 1). RV global strains (global longitudinal strain, GLS; global circumferential strain, GCS; global radial strain, GRS) were measured similarly on short and long-axis cine images. LGE was qualitatively assessed using complete short-axis coverage. Once the two observers held opposing standpoints on LGE existence; they negotiated and ultimately reached a consensus.

### CMR presentations

RV and LV involvements were defined based on CMR findings. RV involvement was identified by the presence of 1 or more following presentations: RVEDVI ≥ 110 mL/m^2^ in males or ≥ 100 mL/m^2^ in females or RVEF < 45%; regional wall motion abnormalities (akinesia or dyskinesia); RV fat infiltration (intramyocardial hyperintensity in fast spin echo or India ink artifact in steady-state free precession), RV late gadolinium enhancement (LGE). LV involvement was suggested if 1 or more following presentations were observed: LV ejection fraction (LVEF) < 50%, LV wall motion abnormalities, LV fat infiltration, LV LGE.

Patients were divided into four groups according to CMR presentations as (1) RV dominant group: with RV involvement and without LV involvement; (2) biventricular group: with both RV and LV involvement; (3) LV dominant group: with LV involvement and without RV involvement; and (4) negative group: with neither RV nor LV involvement.

### Follow-up and clinical outcome

Follow-up was performed annually in all patients for up to a median of 5 years (25th–75th: 4 to 5.5 years) after the CMR examination by one physician who was blinded to CMR data. All feedback was acquired via telephone or from periodic ambulatory visits. The endpoint was defined as the combination of SCD, aborted cardiac arrest, and appropriate implantable cardioverter-defibrillator (ICD) intervention. ICD interventions were considered appropriate when performed due to lethal arrhythmias: ventricular tachycardia above the defaulted cutoff of the ICD (12 intervals at > 180 beats/min) or ventricular fibrillation. The referring physician confirmed the appropriateness of the intervention through a complete inquiry into the ICD performance.

### Statistical analysis

Categorical variables were presented as frequencies (percentage), and continuous variables as the mean ± standard deviation (SD) or as median (IQR). The normality of data was tested by the Kolmogorov–Smirnov test. The chi-square test or Fisher exact test compared categorical variables. Continuous variables were compared by the Student’s *t*-test or Mann–Whitney *U* test. Inter- and intra-observer variability for RA strain and strain rates was assessed by interclass correlation coefficient (ICC).

Cox regression analysis was performed to weigh the impact of all clinical and CMR parameters. Given the number of events available and the high correlation between variables, we selected 5 variables associated with the endpoint at the level of *p* < 0.05 after univariate analysis to undergo a multivariable backward-conditional selection (covariables included 5-year ARVC risk score, RV GLS, RVEDVI, right atrial ejection fraction [RAEF], LV Involvement). After the selection, the two factors (5-year ARVC risk score and LV Involvement) with a relatively higher hazard ratio (HR) filtered at the *p* < 0.05 level made up multivariable baseline model (model 1). RA strain and strain rates were then separately added into model 1 to perform additional multivariable analyses. RA reservoir strain (model 2: model 1 plus reservoir strain) and booster strain (model 3: model 1 plus booster strain) were established as independent predictors of the endpoint. The incremental values of reservoir strain and booster strain to clinical and other CMR predictors for adverse events were shown in the form of C-index, net reclassification index (NRI), and Integrated Discrimination Improvement (IDI). Time-dependent receiver operating characteristic (ROC) analysis was used to compare the prognostic value of the 3 models within 5 years. Cubic spline analysis was used to dichotomize the variables with HR of 1.00 as the cutoff value to perform Kaplan–Meier analysis.

Statistical analysis was performed using R (version 1.3) and the IBM SPSS statistics software package (v. 24.0, Armonk, NY). *p* < 0.05 indicated statistical significance.

## Results

### Study population

**Fig. 2:**
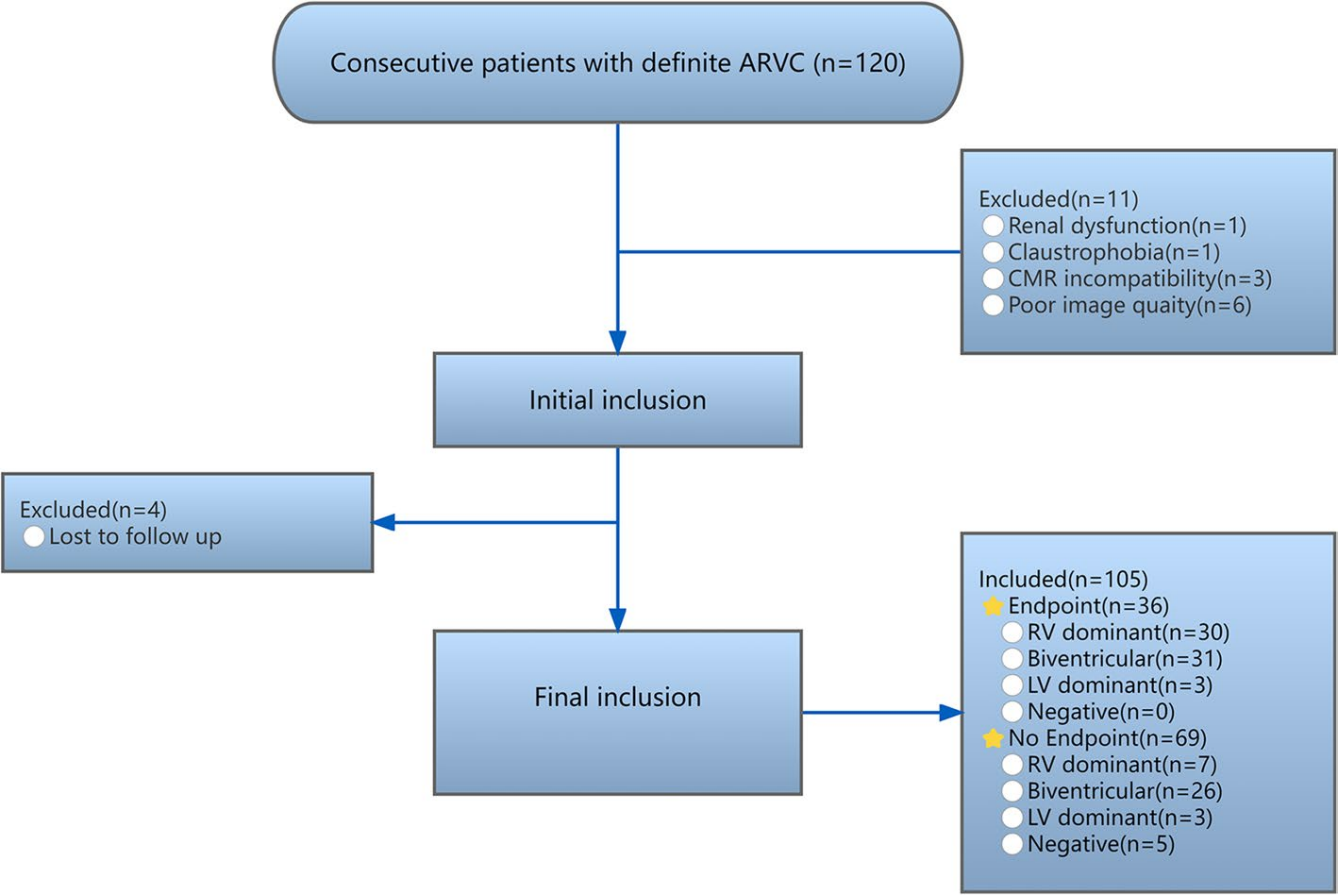
Study inclusion flow-chart of the patients. RV dominant group: patients with RV involvement and without LV involvement; biventricular group: patients with both RV and LV involvement; LV dominant group: patients with LV involvement and without RV involvement; negative group: patients with neither RV nor LV involvement

**Fig. 3:**
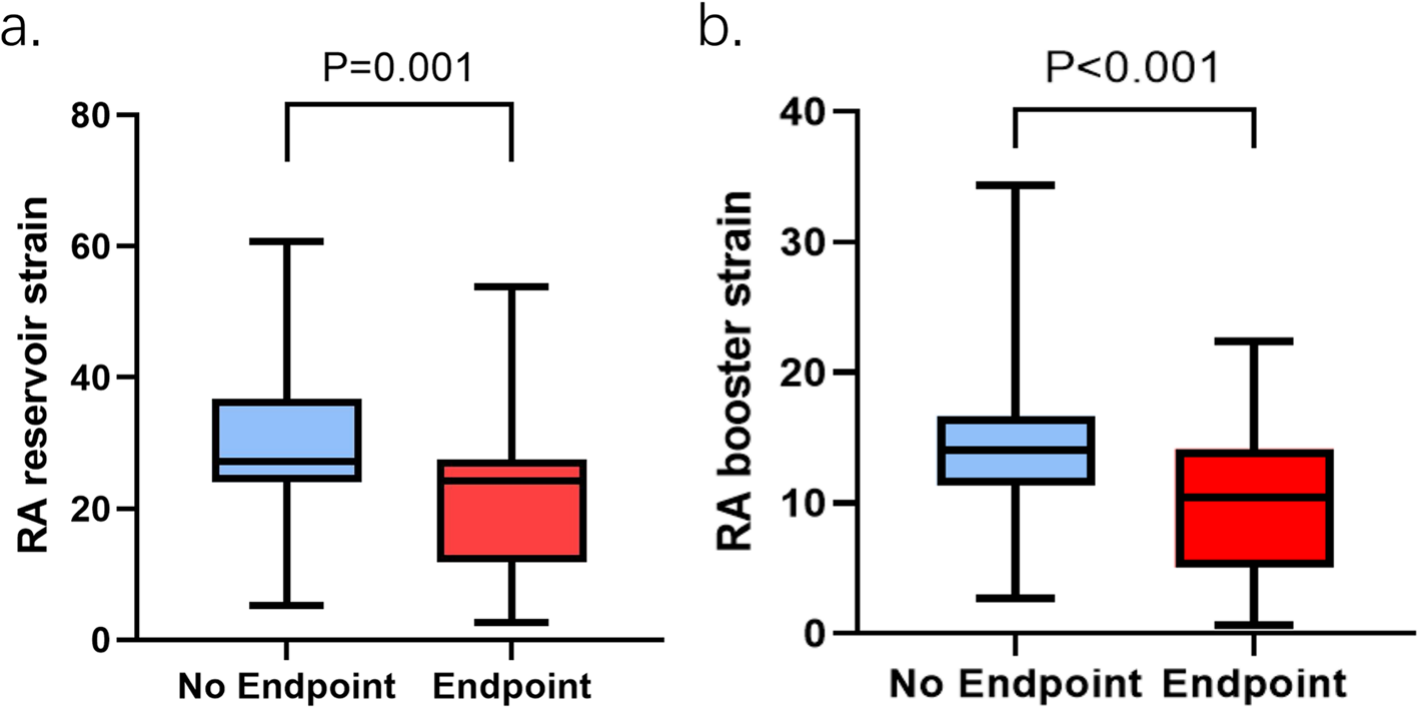
RA reservoir and booster strain in ARVC patients with and without an endpoint. Patients with endpoint had a significantly reduced RA reservoir (**a**) and booster strain (**b**)

As is shown in the study diagram (Fig. 2), among 120 patients who were initially prospectively included, 15 were excluded due to the following reasons: 1 for renal function impairment, 1 for claustrophobia, 3 for CMR incompatible devices, and 6 for poor image quality, after a median follow-up of 60 months (interquartile range, 48–66 months), 4 participants were lost. Finally, 105 participants with a mean age of 48 years were included. Among them, 68 were male (65%). 36 participants (34%) reached the endpoint: 31 (86%) for appropriate implantable ICD intervention, 1 (3%) for sudden cardiac death, and 4 (11%) for aborted cardiac arrest. 37 (35%) were divided into the RV dominant group, 57 (54%) suffered from biventricular involvement, 6 (6%) belonged to the LV dominant group, and 5 (5%) had negative CMR presentation. Participants with endpoint more frequently experienced recent cardiac syncope, non-sustained ventricular tachycardia (NVST), more premature ventricular contraction (PVC) count during 24 h, and greater T-wave inversion (TWI) in anterior and inferior leads compared with participants without an endpoint. They also tended to display lower RV ejection fraction, worse RV strain, and larger RV size. In addition, LV ejection fraction was lower in participants with endpoints, while the prevalence of late gadolinium enhancement (LGE), wall motion abnormality (WMA), and fatty infiltration in LV were higher (Table 1). As for RA parameters, beyond reduced RV ejection fraction, there were differences in all strains, including reservoir (Fig. 3a), conduit, and booster strain (Fig. 3b) and strain rates between participants with and without endpoint (Table 1). The inter-and intra-observer correlation coefficient values for RA strain and strain rate were all at an excellent level (Table S1). Compared with healthy participants, patients with ARVC had significantly impaired RA parameters (all *p* < 0.05) (Table S2).

**Table 1:** Characteristics of patients with and without major endpoint

	**No endpoints**	**Endpoints**	***p* value**
**Demographics**			
Age (years)	44 ± 16	42 ± 20	0.504
Men	47 (68)	21 (58)	0.319
Hypertension	16 (23)	7 (19)	0.660
Diabetes	4 (6)	2 (6)	0.664
**Task force criteria**			
Echocardiographic TF major criterion	26 (38)	13 (36)	0.874
Echocardiographic TF minor criterion	22 (32)	12 (33)	0.880
RV angiography	58 (84)	29 (81)	0.651
ECG major repolarization criterion	17 (25)	13 (36)	0.217
ECG minor repolarization criterion	16 (23)	9 (25)	0.836
ECG major depolarization criterion	4 (6)	2 (6)	0.664
ECG minor depolarization criterion	32 (46)	18 (50)	0.724
Arrhythmias major criterion	19 (28)	7 (19)	0.362
Arrhythmias minor criterion	31 (45)	19 (53)	0.445
Family history	15 (22)	9 (25)	0.706
CMR major criterion	50 (72)	27 (75)	0.780
CMR minor criterion	12 (17)	2 (6)	0.770
**Clinical presentation**			
Recent cardiac syncope	5 (7)	8 (22)	0.027
NVST	28 (41)	23 (64)	0.023
24-h PVC count	983 (385–2529)	2580 (475–4422)	0.010
Leads with anterior and inferior TWI	2 (1–3)	3 (2–4)	0.001
5-yr ARVC risk score	0.22 (0.11–0.38)	0.35 (0.17–0.72)	0.012
**CMR parameters**			
RVEDVI (mL/m^2^)	116.32 (87.21–146.30)	121.69 (90.49–167.16)	0.181
RVESVI (mL/m^2^)	73.78 (51.90–109.57)	87.94 (69.49–135.55)	0.037
RVEF (%)	32.89 ± 12.66	24.86 ± 11.37	0.002
RV GLS (%)	- 8.49 (- 11.45 to - 5.94)	- 5.55 (- 8.45 to - 3.74)	0.003
RV GCS (%)	- 6.72 (- 10.96–3.20)	- 4.97 (- 8.71 to - 1.73)	0.078
RV GRS (%)	19.10 (10.66–30.07)	12.14 (4.38–20.37)	0.008
RV LGE presence	47 (68)	22 (61)	0.472
RV WMA	57 (83)	28 (78)	0.550
RV fatty infiltration	18 (26)	11 (31)	0.627
RAEDVI (mL/m^2^)	43.58 (33.59–59.12)	43.44 (30.47–95.90)	0.466
RAESVI (mL/m^2^)	21.07 (15.76–30.93)	24.64 (16.95–50.91)	0.142
RAEF (%)	49.63 (44.78–55.95)	45.51 (40.89–49.51)	0.009
RA reservoir strain (%)	27.10 (24.00–36.75)	24.20 (11.88–27.50)	0.001
RA conduit strain (%)	14.20 (9.15–19.35)	11.55 (6.18–13.58)	0.004
RA booster strain (%)	14.00 (11.35–16.70)	10.45 (5.05–14.18)	< 0.001
RA reservoir strain rate (sec^-1^)	1.60 (1.10–1.95)	1.10 (0.73~1.50)	< 0.001
RA conduit strain rate (sec^-1^)	- 0.90 (- 1.35 to - 0.70)	- 0.65 (- 0.80 to - 0.43)	< 0.001
RA booster strain rate (sec^-1^)	- 1.60 (- 1.90 to - 1.20)	- 1.30 (- 1.60 to - 0.80)	0.007
**LV involvement analysis**			
LVEF (%)	58.80 (50.35–61.94)	32.07 (22.44–39.31)	< 0.001
LV LGE presence	33 (48)	29 (81)	0.001
LV WMA	20 (29)	26 (72)	< 0.001
LV fatty infiltration	27 (39)	23 (64)	0.016
LV Involvement	34 (49)	29 (81)	0.002

Values are mean ± SD, *n* (%), or median(25th–75th)

*RV* right ventricular, *ECG* electrocardiogram, *NVST* nonsustained ventricular tachycardia, *PVC* premature ventricular contraction, *TWI* T-wave inversion, *RVEDVI* right ventricular end-diastolic volume index, *RVESVI* right ventricular end-systolic volume index, *RVEF* right ventricular ejection fraction, *GLS* global longitudinal strain, *GCS* global circumferential strain, *GRS* global radial strain, *RAEDVI* right atrial end-diastolic volume index, *RAESVI* right atrial end-systolic volume index, *LGE* late gadolinium enhancement, *WMA* wall motion abnormalities, *RAEF* right atrial ejection fraction, *LV* left ventricular, *LVEF* left ventricular ejection fraction. Five-year ARVC risk score, 1 − 0.8396exp (LP) where LP = 0.49*sex − 0.022*age − 0.66*history of recent cardiac syncope + 0.81*history of NSVT + 0.17*ln(24 h PVC count) + 0.11*Sum of anterior and inferior leads with TWI − 0.025*RVEF [20]

### Variable selection for models

The 5-year ARVC risk score, RV GLS, and LV involvement were identified as independent predictors by the backward conditional selection procedure. To eradicate overfitting, we singled out two variables (5-year ARVC risk score and LV involvement) with higher HR and known prognostic value constituting a small subgroup of predictive factors of endpoint (model 1). Therefore, for multivariable analysis, all RA strain and strain rates were separately included in model 1. In addition, RA reservoir strain and booster strain were factored in when establishing final multivariable analysis models (models 2 and 3) as they showed significant independence after adjustment for model 1. RA conduit strain and all RA strain rates had no evident prognostic value of adverse outcomes in ARVC patients (Table 2).

**Table 2:** Univariable analysis and multivariable analysis for endpoints

	**Univariable analysis**		**Multivariable backward conditional selection**	
	HR (95% CI)	*p*-value	HR (95% CI)	*p*-value
**Demographics**				
Age (years)	0.992 (0.973–1.011)	0.402		
Men	0.476 (0.404–1.525)	0.476		
Hypertension	0.678 (0.296–1.553)	0.357		
Diabetes	1.403 (0.331–5.938)	0.646		
**Clinical presentation**				
Recent cardiac syncope	2.168 (0.986–4.766)	0.054		
NVST	2.805 (1.404–5.603)	0.003		
24-h PVC count per 0.01	1.026 (1.012–1.040)	< 0.001		
Leads with anterior and inferior TWI	1.422 (1.168–1.732)	< 0.001		
5-year ARVC risk score	12.403 (3.664–41.982)	< 0.001	6.441 (1.745–23.777)	0.005
**CMR parameters**				
RVEDVI (mL/m^2^)	1.008 (1.000–1.015)	0.036		
RVESVI (mL/m^2^)	1.011 (1.004–1.019)	0.004		
RVEF (%)	0.954 (0.925–0.983)	0.002		
RV GLS (%)	1.057 (1.006–1.111)	0.029	1.073 (1.006–1.144)	0.032
RV GCS (%)	1.034 (0.988–1.083)	0.147		
RV GRS (%)	0.967 (0.938–0.997)	0.033		
RV LGE presence	0.777 (0.397–1.520)	0.461		
RV WMA	0.840 (0.382–1.847)	0.665		
RV fatty infiltration	1.122 (0.552–2.281)	0.751		
RAEDVI (mL/m^2^)	1.002 (0.996–1.008)	0.603		
RAESVI (mL/m^2^)	1.003 (0.995–1.011)	0.509		
RAEF (%)	0.972 (0.948–0.997)	0.028		
LV involvement	3.162 (1.380–7.247)	0.007	2.637 (1.072–6.491)	0.035

5-year ARVC risk score, RV GLS, RVEDVI, RAEF, and LV involvement were included in multivariate analysis after selection

*HR* hazard ratio, *CI* confidence interval; other abbreviations as in Table 1

### Prognostic value of RA strain

**Fig. 4:**
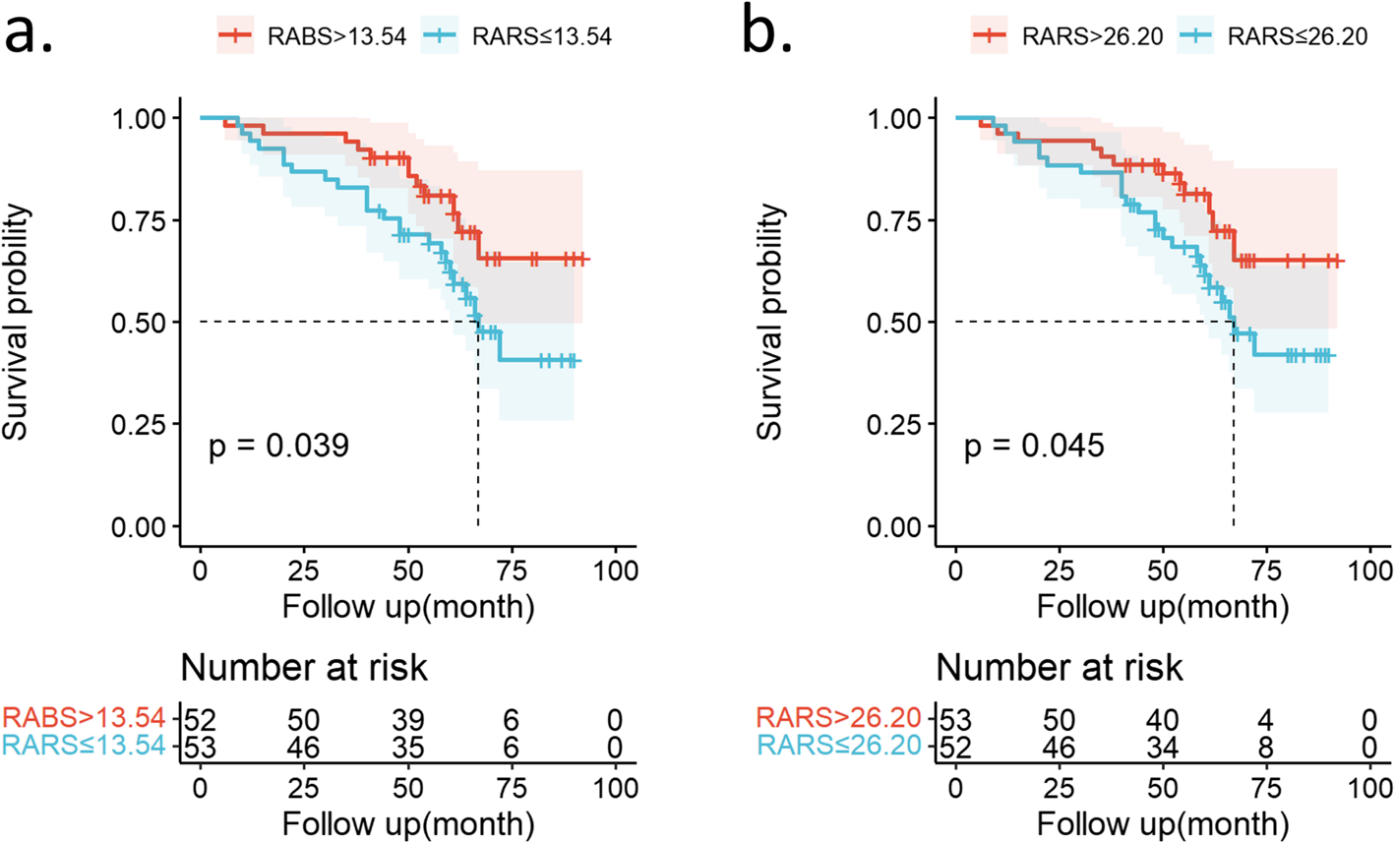
Kaplan–Meier curves represent the survival of participants who were free of endpoint. Participants with RA booster strain (RABS) of ≤ 13.54% (**a**) and right atrial reservoir strain (RARS) of ≤ 26.20% (**b**) displayed significantly higher risk of endpoint

AUC, sensitivity, and specificity of right ventricular and atrial parameters for end-point correlation are demonstrated in Table S3. After backward-conditional multivariable selection, 5-year ARVC risk score, RV GLS, and LV involvement were identified as independent risk factors for the endpoint in ARVC patients. In the final multivariable analysis (Table 3), RA reservoir strain (hazard ratio, 0.956; 95% confidence interval: 0.927, 0.986; *p* = 0.005) and RA booster strain (hazard ratio, 0.906; 95% confidence interval: 0.851, 0.964; *p* = 0.002) (models 2 and 3; Table 3) were determined as independent predictors of the endpoint of ARVC after adjustment for 5-year ARVC risk score and LV Involvement. 5-year ARVC risk score (hazard ratio, 5.940; 95% confidence interval: 1.589, 22.208; *p* = 0.028) remained independently associated with the ARVC endpoint. In Kaplan–Meier survival analysis, patients with booster strain < 13.54% (Fig. 4a) or RA reservoir strain < 26.20% (Fig. 4b) showed a higher risk of endpoint (both log-rank *p* < 0.05).

**Table 3:** Multivariable Cox proportional hazards model for major adverse cardiac event risk by using selected clinical variables and right atrial reservoir and booster strains

	Model 2: Model 1 with RA reservoir strain		Model 3: Model 1 with RA booster strain	
	HR (95% CI)	*p*-value	HR (95% CI)	*p*-value
5-year ARVC risk score	6.001 (1.622–22.206)	0.007	5.940 (1.589–22.208)	0.028
LV involvement	2.038 (0.851–4.884)	0.110	2.191 (0.917–5.233)	0.077
RA reservoir strain (%)	0.956 (0.927–0.986)	0.005		
RA booster strain (%)			0.906 (0.851–0.964)	0.002

Model 1 consists of 5-year risk score and LV involvement

### Incremental value of RA strain

**Fig. 5:**
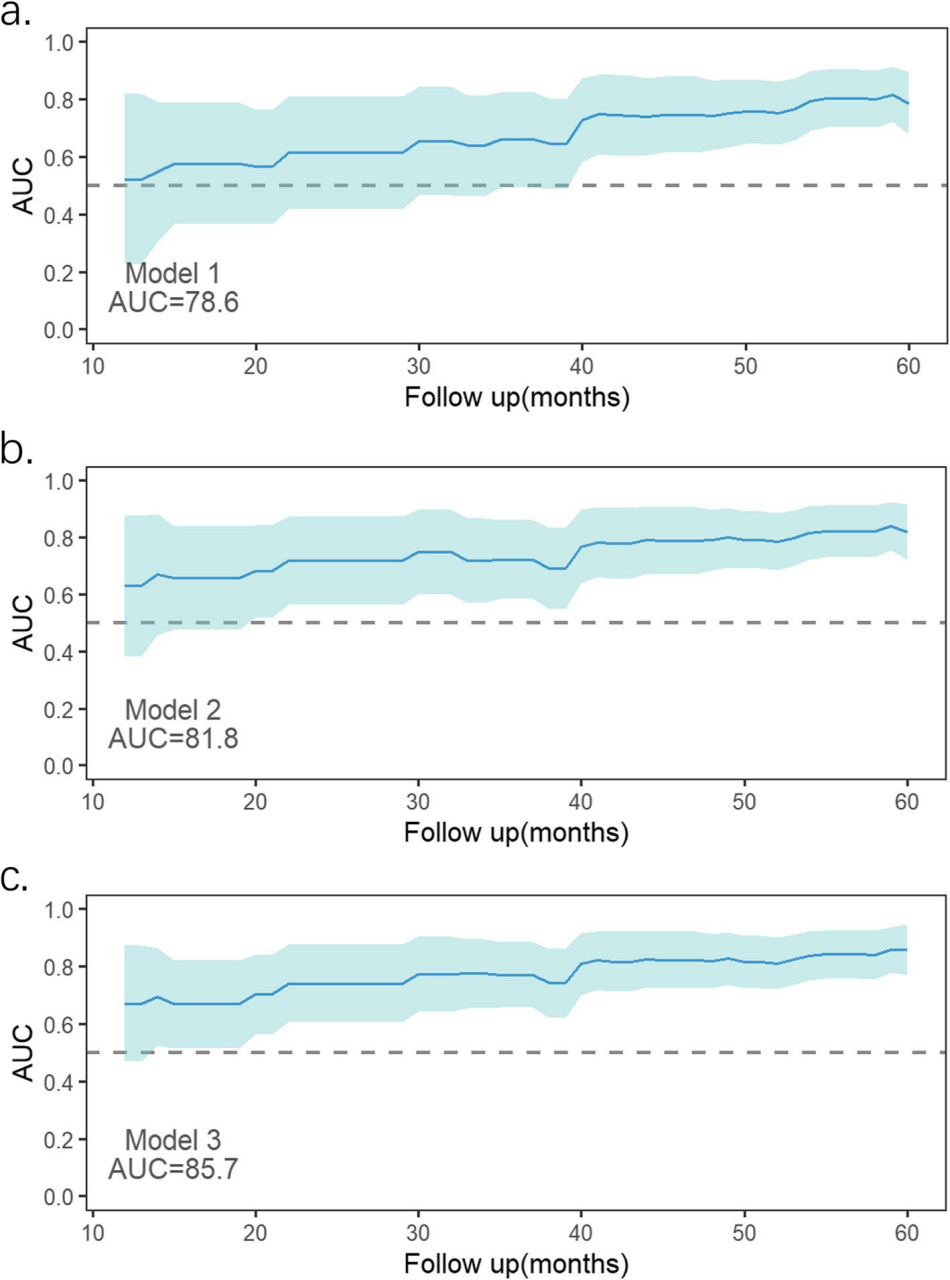
Time-dependent receiver operating characteristic analysis for 3 models. A model with right atrial booster strain had the greatest integrated areas under the receiver operating characteristic curve (AUCs)

C index increased from 0.707 to 0.748 (*p* = 0.004) when RA reservoir strain was added to independent (5-year ARVC risk score and LV Involvement) ARVC risk predictors (model 1), indicating a larger predictive capability for the endpoint. Similarly, adding RA booster strain provided prognostic accuracy for the endpoint (C index, 0.769 vs. 0.707; *p* < 0.001). NRI (0.317, *p* = 0.044) and IDI (0.125, *p* = 0.006) were also indicative of the same tendency when booster strain was included in model 1, while when referring to reservoir strain, NRI showed no statistical significance (Table 4). Results of time-dependent ROC analysis on 3 models predicting endpoint are shown in Fig. 5. Models with RA reservoir and booster strain exhibited greater integrated areas under the ROC curve for predicting endpoints than the initial model (model 1). Also, the booster strain model had the highest prognostic value straightforwardly.

**Table 4:** Model discrimination and reclassification improvement for endpoint after addition of RA strain to the multivariable baseline model

	C-index (95% CI)	*p*-value	NRI (95% CI)	*p*-value	IDI (95% CI)	*p*-value
Model 1	0.707 (0.617–0.797)					
Model 2	0.748 (0.668–0.828)	0.004	0.288 (- 0.068–0.557)	0.114	0.098 (0.021–0.193)	0.006
Model 3	0.769 (0.696–0.842)	< 0.001	0.317 (0.006–0.606)	0.044	0.125 (0.031–0.237)	0.006

*NRI* net reclassification index, *IDI* Integrated Discrimination Improvement

### LV involvement and RA strain

LV involvement was observed in 63 patients, 57 of whom also suffered RV involvement. Patients with LV involvement showed significantly decreased RA conduit strain than those without LV involvement. However, the two groups had no significant differences in other RA strain and strain rate indexes (Table S4). RA conduit strain and conduit strain rate also vary among CMR presentations (RV dominant, biventricular, LV dominant, MRI negative). More details are shown in Table S5.

## Discussion

In this prospective study, we evaluated the prognostic value of RA strain indices derived from CMR images in patients with ARVC after a median follow-up time of 5 years with the following findings. First, patients with ARVC had significantly reduced RA strain and strain rates (all *p* < 0.05) compared with healthy participants. Second, participants with lower RA reservoir and booster stains were associated with a significantly higher risk of adverse cardiac events. Third, RA reservoir strain (HR = 0.956, *p* < 0.05) and RA booster strain (HR = 0.906, *p* < 0.05) resulted as independent predictors of endpoint after adjusting for established clinical and CMR markers of ARVC risk. Finally, RA reservoir strain and booster strain provided greater prognostic value to conventional predictors in predicting the endpoint of ARVC, especially booster strain (C statistic comparing models, 0.769 vs. 0.748; *p* < 0.05). Based on the results, we suggested right atrial strain, derived from non-invasive imaging examination, as a new biomarker for early clinical therapy in medication and ICD implantation in order to moderate the onset of adverse endpoints in ARVC patients.

Cardiac magnetic resonance imaging analysis has a diagnostic role in ARVC by evaluating RVEDVI, RVEF, and RV wall motion abnormalities and providing prognostic value for patients with ARVC. CMR-based parameters have an important role in risk stratification. RV structural and functional indices, such as increased RV size, reduced RV strain, and RV LGE, show prognostic importance in ARVC risk [10, 21–23]. Moreover, LV and RA involvement, with a high prevalence in ARVC patients, can be identified through CMR images. LV involvement is considered to account for ARVC adverse ventricular outcomes [8, 24]. RA volume and RA strain were found to differ significantly between ARVC patients and healthy participants. According to a large cohort study, RAEF and RA conduit strain rate were recognized as predictors of atrial arrhythmias in ARVC patients during long-term follow-up [15]. In their single-center retrospective design, Anwer et al. [16] explored the prognostic significance of RA strain in ARVC using speckle-tracking echocardiographic, finding that RA reservoir and booster strain were related to adverse cardiovascular outcomes at a 5-year follow-up. However, RA strain measurement by speckle tracking is limited by the low signal-to-noise ratio and the thin atrial wall. Several different studies have confirmed the feasibility and reproducibility of CMR-based tissue tracking for assessing atrial dimensions, function, and deformation [20, 25]. Hence, our study mainly focused on strain using tissue tracking derived from CMR cine images with better quantification to obtain increased reliability and reproducibility.

Previous studies identified dilated RV volume, impaired RV function, and LV involvement as risk predictors for adverse cardiac events in ARVC [24, 26]. Consistent with these studies, participants with RV dilatation, reduced RV function, and LV involvement had a higher possibility of reaching the endpoint in our population. Furthermore, the present study assessed RA deformation and provided evidence that RA reservoir and booster strain and 5-year risk score were strong independent predictors of adverse cardiac events at long-term follow-up, which was in line with previous studies [14, 16, 27]. The 5-year ARVC risk score, RV GLS, and LV involvement are all strongly associated with cardiac events in ARVC [21, 24, 27], while the results of the statistical selection algorithm indicated that RA reservoir strain and booster strain provide additional prognostic information in ARVC beyond RV GLS and LV involvement. In addition, our results also suggested that RA reservoir and booster strain provided incremental prognostic capability over clinical characteristics and CMR findings, which was not explored in previous studies. Hence, impaired RA strain may indicate worse outcomes and should be considered useful adjuncts to establish ARVC risk prediction. It is worth mentioning that the RA booster function showed higher predictive value for the ARVC endpoint than reservoir and conduit function did in this population. RA booster function represents intrinsic RA contractility and serves during RA active emptying period. In contrast, reservoir and conduit function, though reflect atrial relaxation, are influenced by ventricular systolic and diastolic performance in part [28]. In ARVC patients, RA booster functional impairment may mainly be caused by RA fibro-fatty replacement. RV strain showed no additional prognostic value according to a recent report [21]. By contrast, our population, with a longer follow-up time and higher event rate, was older and had a higher proportion of males. Especially, we had significantly lower RV function and RV strain. The worse cardiac condition of our population may be the cause of the difference of the results. Patients with LV involvement were detected with significantly lower RA strain and strain rates compared with patients without LV involvement in our study. Also, this was the first study that discovered the potential correlation between LV involvement and RA conduit function. With regard to LV parameters, LV dysfunction has been confirmed to have independent prognostic value in predicting ARVC endpoints [8]. Herein, we found a significant association between endpoint with LV wall motion abnormalities, fat infiltration, and LGE in univariate Cox analyses. However, according to a high correlation among these three parameters, we used LV involvement to present the whole LV impairment. As a combination, LV involvement had decreased significantly in predicting endpoints than respective ones and may conceal prognostic value. However, in a previous study, LV involvement was the best independent combined predictor [8]. The observed difference could be due to the following reasons: in this study, patients with endpoint tended to have LV wall motion abnormalities, fat infiltration, and LGE manifestations simultaneously, whereas patients without endpoint had sporadic LV presentation. When a general metric was used to differentiate the two groups, no endpoint group had a relatively higher volume of patients with LV involvement, causing the weight of LV involvement to decrease in the prediction of ARVC endpoint.

Our study has several limitations. First, the study sample size was relatively small due to the scarcity of ARVC disease, and the number of endpoints also limited the accuracy of statistical analysis. The cutoff value of strain parameters obtained from cubic spline analysis may not represent the truth. Additionally, there is a possibility of overfitting in multivariable survival analysis. Thus, a larger population with a higher event rate is needed for our future analysis. Second, due to the absence of RV two-chamber view, RA strain data were merely derived from CMR four-chamber long-axis views. Future studies based on two- and four-chamber CMR cine images should further evaluate the validity of strain parameters. RA strain analysis was based on tissue-tracking measurement of CMR image postprocessing software, while the credibility of the method is still uncertain. A recent study reported lower reproducibility in strain rate assessment compared with global strain parameters, implying that this might be introduced by the limited temporal resolution of cine CMR images [29]. Hence, tissue-tracking algorithms need modification to improve the reproducibility of RA strain rate analysis. Furthermore, RV LGE was confirmed as a predictor of all-cause mortality and major adverse cardiac events in ARVC, according to a recent meta-analysis [23]. Conversely, in our study, the prognostic value of RV LGE was concealed, which may be explained by the thinness of RV causing variability in measurement and lack of quantification of LGE area. Further studies are needed to evaluate the incremental value of RA strain with RV LGE complement.

In conclusion, participants with impaired RA reservoir and booster strain had a higher risk of adverse cardiac events in ARVC. As independent predictors, RA reservoir and booster strain provided incremental prognostic value for clinical and other CMR predictive factors in ARVC and should therefore be considered as relevant factors for a comprehensive assessment of risk prediction in ARVC.

### Supplementary Information

**Additional file 1: Table S1.** Intra-observer and inter-observer reproducibility for RA strain and strain rate. **Table S2.** Right Atrial Parameters Analysis in ARVC Group and Healthy Group. **Table S3.** AUC, Sensitivity, Specificity, PPV, and NPV of Right Ventricular and Atrial Parameters for Predicting Endpoint correlation. **Table S4.** Differences between Patients with or without Left Ventricular Involvement. **Table S5.** CMR Parameters of Patients With Different CMR Presentations.

## Data Availability

The datasets used and/or analyzed during the current study are available from the corresponding author on reasonable request.

## Abbreviations

CI: Confidence interval
ECG: Electrocardiogram
EDVI: End-diastolic volume index
EF: Ventricular ejection fraction
ESVI: End-systolic volume index
GCS: Global circumferential strain
GLS: Global longitudinal strain
GRS: Global radial strain
HR: Hazard ratio
ICC: Interclass correlation coefficient
IDI: Integrated Discrimination Improvement
LGE: Late gadolinium enhancement
LV: Left ventricular
NRI: Net reclassification index
NVST: Nonsustained ventricular tachycardia
PVC: Premature ventricular contraction
RA: Right atrial
RV: Right ventricular
TWI: T-wave inversion
WMA: Wall motion abnormalities
