# Supplementary material for: Prognostic value of right atrial strains in arrhythmogenic right ventricular cardiomyopathy
Source: Insights Imaging. 2024 Feb 27;15:58. doi: 10.1186/s13244-024-01630-x (PMC10899550; doi:10.1186/s13244-024-01630-x)
Supplement: Supplementary file 1 — Additional file 1: Table S1. Intra-observer and inter-observer reproducibility for RA strain and strain rate. Table S2. Right Atrial Parameters Analysis in ARVC Group and Healthy Group. Table S3. AUC, Sensitivity, Specificity, PPV, and NPV of Right Ventricular and Atrial Parameters for Predicting Endpoint correlation. Table S4. Differences between Patients with or without Left Ventricular Involvement. Table S5. CMR Parameters of Patients With Different CMR Presentations. [file 13244_2024_1630_MOESM1_ESM.pdf]

Prognostic value of right atrial strains in arrhythmogenic right ventricular cardiomyopathy

ELECTRONIC SUPPLEMENTARY MATERIAL

Table S1: Intra-observer and inter-observer reproducibility for RA strain and strain rate

	**Intra-observer**		**Inter-observer**	
	**ICC (95% CI)**	**P value**	**ICC (95% CI)**	**P value**
RA reservoir strain (%)	0.98(0.97~0.98)	<0.001	0.97(0.96~0.98)	<0.001
RA conduit strain (%)	0.97(0.95~0.98)	<0.001	0.97(0.95~0.98)	<0.001
RA booster strain (%)	0.96(0.94~0.97)	<0.001	0.96(0.94~0.97)	<0.001
RA reservoir strain rate (sec-1）	0.96(0.94~0.97)	<0.001	0.95(0.93~0.97)	<0.001
RA conduit strain rate (sec-1）	0.96(0.94~0.97)	<0.001	0.95(0.93~0.97)	<0.001
RA booster strain rate (sec-1）	0.96(0.95~0.98)	<0.001	0.95(0.93~0.97)	<0.001

Table S2: Right Atrial Parameters Analysis in ARVC Group and Healthy Group

	Normal (n=50)	ARVC (n=105)	P Value
Age	34.50(29.25~48.25)	48.00(29.00~57.00)	0.102
Male	26(52)	68(65)	0.128
RAEF (%)	54.94(45.20~61.21)	47.37(43.64~53.54)	0.001
RA reservoir strain (%)	42.90(34.25~52.13)	26.30(21.30~32.10)	<0.001
RA conduit strain (%)	27.30(20.48~37.18)	12.60(8.45~17.45)	<0.001
RA booster strain (%)	15.70(11.70~19.60)	13.60(9.95~15.90)	0.004
RA reservoir strain rate (sec^{-1})	2.25(1.80~3.03)	1.50(1.00~1.80)	<0.001
RA conduit strain rate (sec^{-1})	-2.50(-3.20~-1.68)	-0.80(-1.20~-0.60)	<0.001
RA booster strain rate (sec^{-1})	-1.95(-2.83~-1.40)	-1.50(-1.85~-1.10)	<0.001

Table S3: AUC, Sensitivity, Specificity, PPV, and NPV of Right Ventricular and Atrial Parameters for Predicting Endpoint correlation

	AUC	Sensitivity, %	95%CI	Specificity, %	95%CI	+LR	-LR	P-Value	cut off
5-yr ARVC risk score	0.650	30.56	16.3 - 48.1	95.65	87.8 - 99.1	7.03	0.73	0.0086	0.62
RVEDVI (mL/m2)	0.580	33.33	18.6 - 51.0	89.86	80.2 - 95.8	3.29	0.74	0.2083	158.51
RVESVI (mL/m2)	0.624	75.00	57.8 - 87.9	49.28	37.0 - 61.6	1.48	0.51	0.0411	71.86
RVEF (%)	0.693	63.89	46.2 - 79.2	75.36	63.5 - 84.9	2.59	0.48	0.0005	23.81
RAEF (%)	0.656	61.11	43.5 - 76.9	71.01	58.8 - 81.3	2.11	0.55	0.0052	46.00
RA reservoir strain (%)	0.707	94.44	81.3 - 99.3	40.58	28.9 - 53.1	1.59	0.14	0.0001	31.30
RA conduit strain (%)	0.670	83.33	67.2 - 93.6	50.72	38.4 – 63.0	1.69	0.34	0.0016	14.03
RA booster strain (%)	0.711	58.33	40.8 - 74.5	78.26	66.7 – 87.4	2.68	0.53	0.0001	10.90
RA reservoir strain rate (sec^{-1})	0.717	91.67	77.5 - 98.2	44.93	32.9 - 57.4	1.66	0.19	<0.0001	1.70
RA conduit strain rate (sec^{-1})	0.716	88.89	73.9 - 96.9	49.28	37.0 - 61.6	1.75	0.23	0.0001	-1.00
RA booster strain rate (sec^{-1})	0.660	33.33	18.6 – 51.0	92.75	83.9 – 97.6	4.60	0.72	0.0071	-1.00
Note: AUC= areas under the receiver operating characteristic curve; LR= likelihood ratio.									

Table S4: Differences between Patients with or without Left Ventricular Involvement

**Demographics**	**Non-LV involvement (n=42)**	**LV involvement (n=63)**	**P-Value**
Age (y)	49(32~60)	45(28~56)	0.165
Men	28(66.67)	40(63.49)	0.739
Hypertension	9(21.43)	14(22.22)	0.923
Diabetes	1(2.38)	5(7.94)	0.230
**Task force criteria**			
Echocardiographic TF major criterion	13(30.95)	26(41.27)	0.284
Echocardiographic TF minor criterion	17(40.48)	17(26.98)	0.146
RV angiography	36(85.71)	51(80.95)	0.526
ECG major repolarization criterion	5(11.90)	25(39.68)	0.002
ECG minor repolarization criterion	13(30.95)	12(19.05)	0.161
ECG major depolarization criterion	0(0.00)	6(9.52)	0.079
ECG minor depolarization criterion	15(35.71)	35(55.56)	0.046
Arrhythmias major	9(21.43)	17(26.98)	0.518

criterion			
Arrhythmias minor criterion	14(33.33)	36(57.14)	0.018
Family history	9(21.43)	15(23.81)	0.776
CMR major criterion	31(73.81)	46(73.02)	0.928
CMR minor criterion	10(23.81)	4(6.35)	0.017
**Clinical presentation**			
Recent cardiac syncope	1(2.38)	12(19.05)	0.014
NVST	13(30.95)	38(60.32)	0.003
24-h PVC Count	699(139~2333)	2159(947~3790)	0.001
Leads with TWI anterior +inferior	2(1~2)	3(1~3)	0.001
5-yr ARVC risk score	0.15(0.09~0.37)	0.34(0.19~0.59)	<0.001
**CMR parameters**			
RVEDVI (mL/m^2)	117.55(89.32~146.53)	120.39(88.79~158.12)	0.484
RVESVI (mL/m^2)	71.93(47.39~104.17)	81.72(65.05~118.14)	0.161
RVEF (%)	31.98(21.54~46.49)	26.83(20.84~36.28)	0.040
RV GLS (%)	-7.72(-10.68~-4.35)	-7.88(-11.00~-5.02)	0.974
RV GCS (%)	-6.67(-11.26~-4.55)	-5.53(-8.51~-1.66)	0.091
RV GRS (%)	18.11(11.69~29.74)	15.97(8.22~27.00)	0.160
RV LGE presence	23(54.76)	46(73.02)	0.054

RV WMA	36(85.71)	49(77.78)	0.310
RV fatty infiltration	8(19.05)	21(33.33)	0.125
RAEDVI (mL/m^2)	43.68(34.68~60.89)	43.11(33.33~67.69)	0.992
RAESVI (mL/m^2)	21.20(15.92~27.74)	25.75(15.72~44.84)	0.289
RAEF (%)	50.06(43.82~55.75)	47.17(43.55~51.89)	0.236
RA reservoir strain (%)	26.70(23.23~37.53)	25.80(20.40~31.30)	0.189
RA conduit strain (%)	15.00(8.88~21.43)	11.80(8.10~14.30)	0.036
RA booster strain (%)	12.55(10.18~15.78)	13.70(9.90~15.90)	0.943
RA reservoir strain rate (sec^{-1})	1.55(1.08~1.83)	1.30(1.00~1.80)	0.221
RA conduit strain rate (sec^{-1})	-0.80(-1.20~-0.60)	-0.80(-1.30~-0.60)	0.839
RA booster strain rate (sec^{-1})	-1.50(-2.00~-1.18)	-1.40(-1.80~-1.00)	0.404
LVEF (%)	58.67(45.34~61.78)	43.87(32.66~55.73)	0.001
LV LGE presence	0	62(98.41)	<0.001
LV WMA	0	46(73.02)	<0.001
LV fatty infiltration	0	50(79.37)	<0.001

Table S5: CMR Parameters of Patients With Different CMR Presentations

	RV dominant(n=37)	Biventricular (n=57)	LV dominant (n=6)	MRI negative (n=5)	P value
RVEDVI (mL/m^2)	120.56(93.69~149.67)‡	126.84(92.14~160.23)‡	74.15(50.35~83.07)§†	93.33(70.24~104.01)	0.002
RVESVI (mL/m^2)	83.41(53.26~109.57)‡	89.25(69.43~120.96)⁎‡	34.26(26.00~48.11)§†	43.07(36.45~52.25) †	<0.001
RVEF (%)	31.57(20.56~41.77) ⁎	25.82(19.79~30.56)⁎‡	46.67(40.81~52.42)†	48.74(46.94~53.54)§†	<0.001
RV GLS (%)	-7.72(-10.49~-4.27)	-7.72(-10.27~-4.67)	-10.08(-14.02~-7.93)	-13.85(-16.15~4.09)	0.280
RV GCS (%)	-5.87(-10.56~-3.73)	-5.35(-8.33~-1.14) ⁎	-9.10(-11.88~-6.76)	-13.70(-15.19~-7.90) †	0.012
RV GRS (%)	15.20(10.66~28.01) ⁎	13.41(7.31~23.60) ⁎	23.00(20.63~39.25)	44.27(34.54~46.36) †§	0.001
RV LGE presence	23(62.16)⁎‡	46(80.70)⁎‡	0(0)§†	0(0)§†	<0.001
RV WMA	36(97.30)⁎‡	49(85.96)⁎‡	0(0)§†	0(0)§†	<0.001
RV fatty infiltration	8(21.63)	21(36.84)	0(0)	0(0)	0.077
RAEDVI (mL/m^2)	43.58(35.94~62.82)	44.08(35.37~75.52)	28.79(25.62~39.11)	47.83(32.72~208.27)	0.156
RAESVI (mL/m^2)	21.07(15.68~29.11)	27.07(17.47~46.86)	12.59(12.13~21.09)	22.70(17.13~135.20)	0.049
RAEF (%)	50.78(43.67~55.79)	47.17(43.40~50.94)	52.00(44.60~57.56)	48.70(38.64~55.21)	0.361
RA reservoir	26.50(23.05~32.65)	25.80(19.40~30.85)	28.20(23.78~41.58)	40.80(22.30~44.40)	0.168

strain (%)					
RA conduit strain (%)	14.70(8.75~17.75)	11.50(8.10~14.17)	13.85(12.83~26.68)	28.30(13.65~30.65)	0.016
RA booster strain (%)	12.80(10.05~16.05)	13.70(9.40~16.05)	12.90(10.65~15.78)	12.50(7.95~14.45)	0.957
RA reservoir strain rate (sec^{-1})	1.50(1.05~1.80)	1.30(1.00~1.80)	1.55(1.28~2.70)	1.80(1.10~2.50)	0.248
RA conduit strain rate (sec^{-1})	-0.70(-0.10~-0.60) ‡	-0.80(-1.10~-0.60) ‡	-1.50(-2.18~-1.00) §†	-1.60(-1.75~-0.90)	0.003
RA booster strain rate (sec^{-1})	-1.50(-1.95~-1.10)	-1.50(-1.80~-1.00)	-1.30(-1.58~-1.28)	-1.70(-2.65~-1.45)	0.479
LVEF (%)	57.85(43.27~61.61)†	43.87(32.07~55.07) §⁎	47.67(38.61~62.72)	60.76(60.51~61.82)†	0.004
LV LGE presence	0(0)†‡	57(100)§⁎	5(83.33)§†	0(0)†	<0.001
LV WMA	0(0)†‡	42(73.68)⁎§	4(66.67)§	0(0)†	<0.001
LV fatty infiltration	0(0)†‡	45(78.95)⁎§	5(83.33)⁎§	0(0)†‡	<0.001

Note: §p<0.05 vs RV dominant; †p<0.05 vs Biventricular; ‡p<0.05 vs LV dominant; ⁎p<0.05 vs MR negative.
